# Secondary Metabolites from Marine-Derived Fungi and Actinobacteria as Potential Sources of Novel Colorectal Cancer Drugs

**DOI:** 10.3390/md20010067

**Published:** 2022-01-12

**Authors:** Elin Julianti, Ikram Ammar Abrian, Marlia Singgih Wibowo, Muhammad Azhari, Nadya Tsurayya, Fauzia Izzati, Ario Betha Juanssilfero, Asep Bayu, Siti Irma Rahmawati, Masteria Yunovilsa Putra

**Affiliations:** 1 School of Pharmacy, Bandung Institute of Technology, Jl. Ganesha No.10, Bandung 40132, Indonesia; ikramammarabrian@gmail.com (I.A.A.); marlia@fa.itb.ac.id (M.S.W.); mazhari@fa.itb.ac.id (M.A.); tsurayya.nadya@gmail.com (N.T.); 2 Research Center for Biotechnology, Research Organization for Life Sciences, National Research and Innovation Agency (BRIN), Jl. Raya Jakarta-Bogor KM 46 Cibinong, Bogor 16911, Indonesia; fauzia.nurul.izzati@brin.go.id (F.I.); ario.betha.juanssilfero@brin.go.id (A.B.J.); asep.bayu@brin.go.id (A.B.); siti.irma.rahmawati@brin.go.id (S.I.R.)

**Keywords:** marine, fungi, actinobacteria, colorectal cancer, cytotoxicity, secondary metabolite

## Abstract

Colorectal cancer is one of the most common cancers diagnosed in the world. Chemotheraphy is one of the most common methods used for the pharmacological treatment of this cancer patients. Nevertheless, the adverse effect of chemotherapy is not optimized for improving the quality of life of people who are older, who are the most vulnerable subpopulation. This review presents recent updates regarding secondary metabolites derived from marine fungi and actinobacteria as novel alternatives for cytotoxic agents against colorectal cancer cell lines HCT116, HT29, HCT15, RKO, Caco-2, and SW480. The observed marine-derived fungi were from the species *Aspergillus* sp., *Penicillium* sp., *Neosartorya* sp., *Dichotomomyces* sp., *Paradendryphiella* sp., and *Westerdykella* sp. Additionally, *Streptomyces* sp. and *Nocardiopsis* sp. are actinobacteria discussed in this study. Seventy one compounds reviewed in this study were grouped on the basis of their chemical structures. Indole alkaloids and diketopiperazines made up most compounds with higher potencies when compared with other groups. The potency of indole alkaloids and diketopiperazines was most probably due to halogen-based functional groups and sulfide groups, respectively.

## 1. Introduction

As the third deadliest cancer, colorectal cancer (CRC) is the fourth type of cancer most diagnosed in the world. By 2018, there were approximately 704,000 new cases of rectal cancer and more than a million new cases of colon cancer diagnosed, summing up to 1.8 million new cases in CRC [1]. Chemotherapies are the most common method for medical treatment of CRC’s patients. Despite the advances in CRC medical treatments, this medication still faces two main issues regarding cancer chemotherapy resistance and the dilemma of specific CRC pharmacotherapy for people who are older, as the most vulnerable subpopulation.

CRC chemotherapy resistance could occur through the variety of gene expressions for related proteins. For instance, the effectiveness of fluorouracil (5-FU) on targeting thymidylate synthase, is highly correlated with the presence of the thymidylate synthase enzyme. The 5-FU effectiveness is also related to its metabolism and degradation by specific proteins such as thymidine phosphorylase, uridine phosphorylase, orotate phosphoribosyl transferase, and dihydropyrimidine dehydrogenase [2]. The expression of the Rac1b gene is overexpressed in the adenocarcinoma form of colon cancer. The overexpression of the Rac1b gene is shown to facilitate chemoresistance against treatment with oxaliplatin or 5-FU through NF-kB signaling [3]. Thus, chemotherapy alone is sometimes insufficient for the treatment of CRC. The mechanism of resistance must be addressed to have a successful treatment outcome.

Additionally, people who are older are the most vulnerable subpopulation of CRC, with a high incidence among elders above 70 years of age. Since each of them has different medical conditions, personalized CRC pharmacotherapy should be addressed and their treatments need to be more specific and should consider the complications of chemotherapy, radiotherapy, or surgery with respect to their frailty, cognitive and functional status, and any related comorbidity that comes along [4].

Exploring alternative sources of active pharmaceutical ingredient (API) for CRC therapy is critical to finding more effective strategies for managing those two issues. Natural compounds from plants have historically occurred in cancer treatment. For examples, irrinotecan (CPT-11) has been used for CRC treatment after the failure of 5-fluorouracil treatment [5,6,7]. Furthermore, andrographolide is a diterpene lactone isolated from the plant *Androphagis paniculata*, which induces the apoptosis of HCT 116 cells in combination with 5-FU [8]. *Curcuma longa* produced curcumin, a polyphenol compound, that induced p21-independent and reduced the secretory level of TNF-α, with significantly improved the quality of life of the patients [9,10]. Marine organisms were also shown to be potential sources for novel compounds against CRC. In addition, a sulfated polysaccharide from brown seaweed, namely fucoidan, displayed apoptosis activity in HCT-116 and HT-29 cells [11]. Moreover, fucoidan has significant activity against CRC, with relatively little side effects for humans in clinical trials [12].

Recently, the exploration of novel anticancer agents against CRCs derived from marine microbiology has attracted much attention. Marine microbes such as fungi, algae, bacteria, and plankton make up to 98% of the biomass within the world’s seas [13]. With the advancement of marine biotechnological techniques, several papers revealed the high potency of mining marine microorganisms for bioactive compounds. Among of them, bacteria and fungi are the prime targets for studies regarding the production of novel bioactive metabolites [14]. In contrast with the oversaturation of API discovery from terrestrial-based microorganisms, marine microorganisms still remain limited in exploration and offer high potency to obtain novel active compounds.

A previous study demonstrated that polyunsaturated fatty acids (PUFAs) from marine microalgae have strong inhibition against the HT-29 cell line [15]. Nowadays, some marine compounds from fungi and actinobacteria show significant activity against CRC cell lines. The research and development on drug discovery based on marine microbes, including for active compounds against CRC, offer a huge potency because these microorganisms could be cultivated as well as modified for large-scale production. Therefore, this paper performs a descriptive study on the discoveries of novel compounds isolated from marine microbes such as fungi and actinobacteria collected from various marine sediments and seawaters. This literature review was conducted by selecting the possibility of becoming alternative agents against CRC concerning cytotoxicity studies.

## 2. Potential Cytotoxic Metabolites from Various Marine Microorganisms against CRC

### 2.1. Marine Fungi

#### 2.1.1. *Aspergillus* sp.

**Figure 1:**
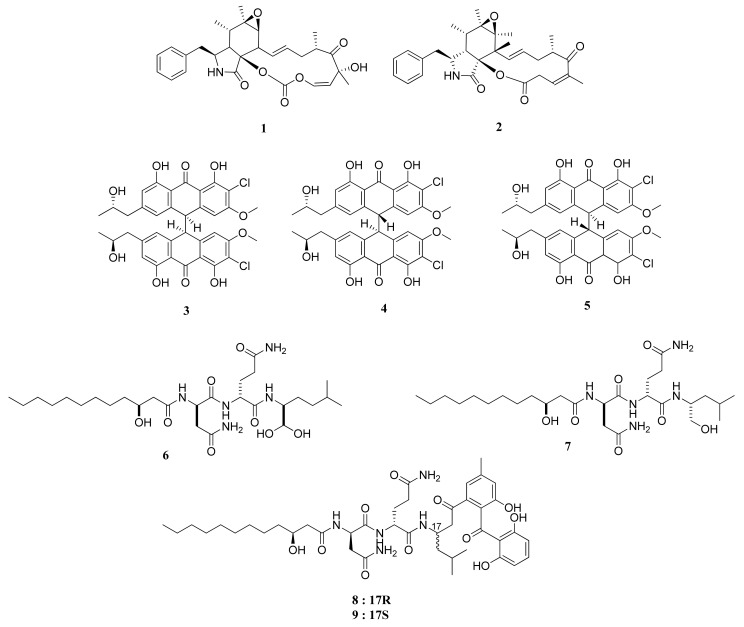
Chemical structures of compounds **1**–**9**.

Rosellichalasin (**1**) and Cytochalasin E (**2**) are two alkaloids isolated from *Aspergillus* sp. nov. F1 (Figure 1). This fungus was obtained from a marine solar saltern in Weihai, China [16]. Both compounds belong to a classification of fungal metabolites known as cytochalasins, which suppress cell division and instigate apoptosis through their actions on a cellular level of degradation and formation disruption of actin filaments [17]. In an MTT assay, these compounds showed moderate and weak activity, with IC_50_ values of 62.3 and 37.3 µM against the RKO CRC cell lines, respectively. The IC_50_ values of cytochalasins were significantly lower than that of ergosterol, which managed to have an IC_50_ value of 3.3 µM [16].

The co-culture from two different growth stages of a new strain of *Aspergillus alliaceus*, coined as the G4 strain, was successfully reported to produce several potent bianthrones: allianthrones A (**3**), B (**4**), and C (**5**). An MTT assay against HCT-116 (CCL-247) CRC cells resulted in IC_50_ values of 9.0, 10.5, and 13.7 μM, respectively, which are more potent than the 11.6% survival rate of HCT-116 cells treated with 250 μM of etoposide as a positive control [18].

The lipopeptides fellutamide F (**6**) and fellutamide C (**7**) were obtained from a symbiotic fungi *Aspergillus versicolor* PF10M of a marine sponge of *Petrosia* sp. from the coastal area of Jeju Island, Korea [19]. These compounds were also reported to be isolated from *Penicillium fellutanum*, a fungus associated with saltwater fish. Compound **6** and its counterpart, compound **7**, exhibited potent cytotoxicity against HCT-15, with ED_50_ values 0.13 and 1.74 µg/mL, respectively. The former substance showed cytotoxic activities higher than that of doxorubicin. It is structurally similar to the hydrated version of fellutamide B [19], found from *P. fellutanum* from the belly of the fish *Apogon endekataenia* located within the waters of the Japanese coast. The compound is also a cytotoxic agent against human epidermoid carcinoma cells KB [20] and is a known proteasome inhibitor [21].

The isolation of secondary metabolites from the fungus *Aspergillus versicolor* Ppf48 yielded several compounds, including asperphenin A (**8**) and asperphenin B (**9**) [22]. Through an SRB assay and tested upon RKO CRC cell line, compounds **8** and **9** showed strong cytotoxic activities, with IC_50_ values of 0.84 and 1.26 µM, respectively. Compounds **8** and **9** hold significance as comparative treatments compared with the positive control of etoposide, with an IC_50_ value of 3.82 μM.

Compound **8** attacks the RKO CRC cells in various ways. Notably, the aryl ketone of C7 of both asperphenins is the primary cause of decreased cell viability. Treatment of this compound against RKO cells is correlated with the cells’ accumulation toward the sub-G1 apoptotic phase. It also increased G2/M phase arrest, induced ROS, and suppressed tubulin polymerization. Moreover, it was observed to induce the decrease in RKO tumor cells volumes in xenograft mice, which can reach lower percentages when combined with irinotecan.

#### 2.1.2. *Penicillium* sp.

**Figure 2:**
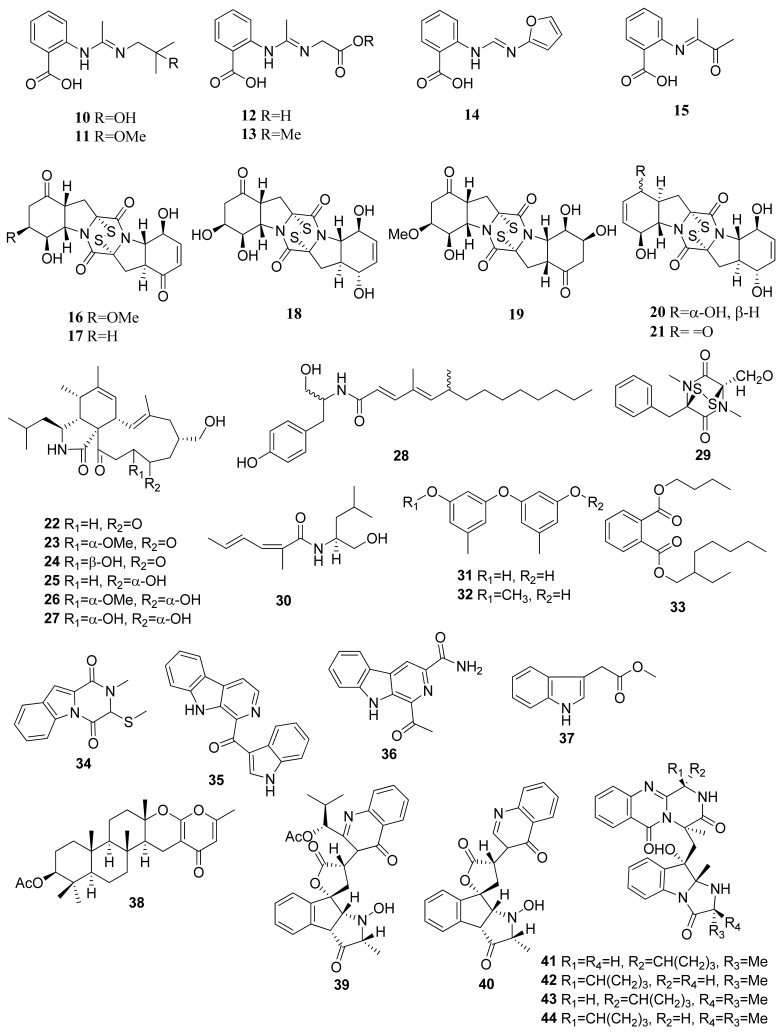
Chemical structures of compounds **10**–**44**.

The isolation of secondary metabolites from *Penicillium paneum* SD-44 obtained the anthranilic acid derivatives penipacids A–E (**10**–**14**) and one synthesized analogue (**15**) (Figure 2). Compounds **10** and **14** revealed inhibitory activity against RKO CRC cells using the MTT assay, obtaining IC_50_ values of 8.4 and 9.7 µM [23]. The IC_50_ values were significantly lower than the positive control agent, fluorouracil, which has an IC_50_ value of 25 µM. Assays of anthranilic acid and its derivatives for their anticancer potential have been studied on several occasions. For instance, an initial in vitro and in vivo effect of anthranilic acid on cancer cells exhibited that the flufenamic acid patterns allow for the presence of functional groups such as (hetero)aryl esters to improve the in vitro tumor cell growth suppression [24].

Brocazines A–F (**16**–**21**) were obtained from endophytic fungus *Penicillium broca* MA-231, which was isolated from marine mangrove *Avicennia marina* tissue from Hainan island, south of Mainland China. Compounds **16** and **17** exhibited IC_50_ values of 2.0 and 1.2 nM, respectively, against SW480 (CCL-228) CRC cells. The results showed high cytotoxic activity when compared with the positive control, cisplatin, having an IC_50_ value of 11.3 µM. Both compounds also showed comparatively stronger activity against NCI-H460 (human large-cell lung carcinoma), SGC-7901 (human gastric carcinoma), and U251 (human glioma cell) cell lines compared with the positive control cefitinib and doxorubicin. The cytotoxic activity was based on the presence of the disulfide bridge within the compound’s structures [25]. Similar diketopiperazines with disulfides can cause cytotoxicity against HCT116 CRC cell lines [26]. The apoptosis caused by diketopiperazine disulfides was shown through Annexin V-propidium iodide staining via flow cytometry. In terms of a dose–response relationship, the number of cells at various stages of apoptosis is directly proportional to concentrations of diketopiperazine disulfides. Treatment of such class of metabolites also correlates with the cleavage of poly (ADP-ribose) polymerase (PARP) and caspase proteins within apoptotic cells as well as with the downregulation of mitochondrial apoptosis allosteric regulators (e.g., Bcl-xL and Bcl-2 anti-apoptotic agents) and the upregulation of pro-apoptotic agents (e.g., Bax).

#### 2.1.3. *Westerdykella* sp.

Seven new alkaloid compounds were isolated from *Westerdykella dispersa* XL602 fungus, collected from the South China Sea marine sediments near Guangzhou, China [27]. The isolated compounds of cytochalasans were named 18-oxo-19,20-dihydrophomacin C (**22**), 18-oxo-19-methoxy-19,20-dihydrophomacin C (**23**), 18-oxo-19-hydroxyl-19,20-dihydrophomacin C (**24**), 19,20-dihydrophomacin C (**25**), 19-methoxy-19,20-dihydrophomacin C (**26**), 19-hydroxyl-19,20-dihydrophomacin C (**27**), and the tyrosine-derivative, gymnastatin Z (**28**). When tested against HT-29 cells through an MTT assay, compounds **25**, **26**, **27**, and **28** displayed moderate inhibitory activity, with IC_50_ values of 49.09, 55.31, 55.48, and 49.1 μM, respectively.

#### 2.1.4. *Paradendryphiella* sp.

The isolation of (−)-(3R, 6R) Hyalodendrin (**29**) was obtained from Paradendryphiella salina PC 362H fungus [28]. This compound was determined using the MTT assay against 15 different type of cancer cell lines, such as SW 48, DLD1, HT 29, HT 29 5FU, HT 29 oxa, HT 29 SN-38, HCT 116, HCT 116 5FU, HCT 116 oxa, HCT 116 SN-38, LS 513, LOVO, RKO, LS174T, and SW 480. Compound **22** revealed strong cytotoxic activity against HCT 116 oxa cells, with an IC_50_ value of 25.7 nM. This compound is characterized by a sulfur-bridged dioxopiperazine that is believed to have an important role in its activity [29]. 

#### 2.1.5. *Dichotomomyces* sp.

Eight compounds were isolated from *Dichotomomyces cejpii* F31-1 fungus associated with the soft coral *Lobophytum crassum* collected from Hainan Sanya National Coral Reef Reserve, China. These compounds were identified as dichotomocej A (**30**), diorcinol (**31**), 3-*O*-methyldiorcinol (**32**), butyl (2-ethylhexyl) phthalate (**33**), dichocerazine A (**34**), pityriacitrin (**35**), stellarine A (**36**), and indolyl-3-acetic acid methyl ester (**37**). All compounds were evaluated against HCT 116. Among them, pityriacitrin (**35**) exhibited a moderate inhibitory effect against HCT116 cells using the SRB colorimetric method, with an IC_50_ value of 35.1 µM [30]. 

#### 2.1.6. *Neosartorya* sp.

The marine se fan-derived fungus *Neosartorya siamensis* KUFC 6349 resulted in eight compounds, namely chevalone C (**38**), nortryptoquivaline (**39**), tryptoquivaline H (**40**), fiscalin A (**41**), epi-fiscalin A (**42**), epi-neofiscalin A (**43**), and epi-fiscalin C (**44**). These compounds tested against HCT116 cells through an MTT assay, and IC_50_ values of 153, 114, 202, 123, 277, 203, and 86 µM were obtained, respectively, for compounds **38**, **39**, **40**, **41**, **42**, **43**, and **44** [31]. In the HCT116 cell line, the compounds **25**, **42**, and **44** slightly decrease cell proliferation (tested at 50 μM), showing cell proliferation inhibitions of 19%, 22%, and 58%, respectively. Compound **44** exhibited significant inhibition of cell proliferation and presented a promising antiproliferative effect (in comparison with doxorubicin 0.1 μM and cell proliferation inhibition of 60%) [31].

### 2.2. Actinobacteria

#### 2.2.1. *Nocardiopsis* sp.

**Figure 3:**
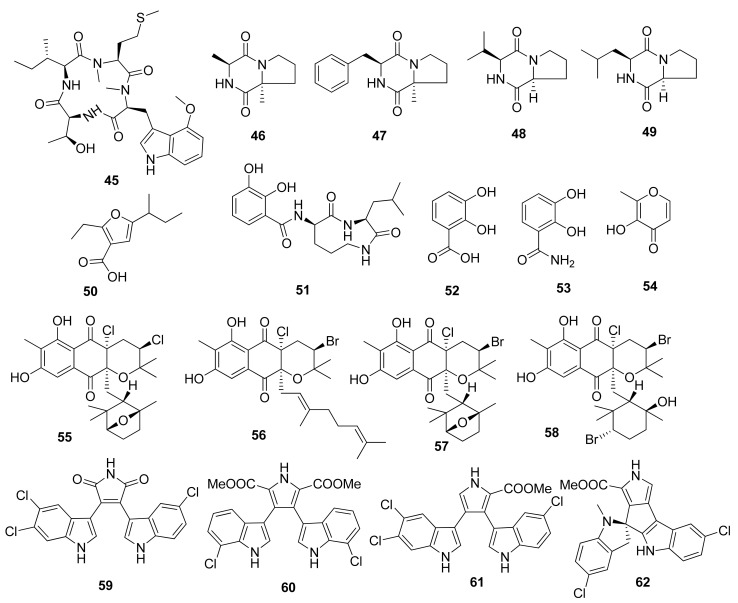
Chemical structures of compounds **45**–**62**.

The discovery of a cyclic tetrapeptide named androsamide (**45**) was obtained from *Nocardiopsis* sp. CNT-189 collected from sediments around the shores on Bahamas (Figure 3) [32]. Its treatment against the Caco-2 and HCT116 cancer cell lines was noted to correlate with the decrease in their viability in a dose-dependent manner. Nevertheless, it did not possess sufficient cytotoxic, with IC_50_ values of 13 and 21 μM, respectively. Nevertheless, it showed apoptosis in Caco-2 CRC cells. Compound **45** inhibited Caco-2 cell migration in a dose-dependent manner. Similarly, the compound was able to decrease EMT transcription factors corresponding to the expression by Caco-2 cells as well as genes related to cell motility in Caco-2, e.g., CAPN1 and RAC2.

#### 2.2.2. *Streptomyces* sp.

Compounds cyclo(Pro-Ala) (**46**), cyclo(Pro-Phe) (**47**), cyclo(Pro-Val) (**48**), and cyclo(Pro-Leu) (**49**) were isolated from marine *Streptomyces* sp. collected from a rhizosphere soil in the mangrove *Avicennia marina* forest of Zhangzhou [33]. The compounds were tested against HCT116 CRC cells through MTT assay, wherein IC_50_ values of 47.6, 32.3, 67.2, and 92.6 µg/mL were obtained, respectively.

The unique furan-type compound (**50**) was isolated from *Streptomyces* sp. VN1 collected from sea sediment offshore at Da Nang Beach, Phu Yen Province, Vietnam. This compound was tested against HCT116 CRC cells through an MTT assay with an IC_50_ value of 123.7 µM [34]. This unique compound was completely elucidated to be 5-(sec-butyl)-2-ethylfuran-3-carboxylic acid. No reference for the nuclear magnetic resonance (NMR) data of compound (**50**) was available. This is the first report of the NMR spectrum and biological activity of the furan-type compound (**50**) produced by a marine *Streptomyces* sp. [34].

Cyclic dipeptides, namely, petrocidin A (**51**), 2,3-dihydroxybenzoic acid (**52**), 2,3-dihydroxybenzamide (**53**), and maltol (**54**), were successfully isolated from symbiotic fungi *Streptomyces* sp. SBT348 from Mediterranean sponge *Petrosia ficiformis*, located off the shores of Pollonia, Milos, Greece [35]. Compounds **51** and **54** were tested against HT29 CRC cells through an MTT assay, and the IC_50_ values were observed at 5.3 and 3.8 µg/mL, respectively. The cytotoxic effect was observed due to the inhibition of overexpressed microsomal prostaglandin E2 synthase-1 on HT-29 cells, which has been discussed in several studies in the literature.

The activity of cyclic dipeptides on CRC cells is comparable with that in a study of the same group of compounds isolated from a soil-based bacterium, *Exiguobacterium acetylicum*, which delves more profound into the anticancer mechanisms. Several cyclic dipeptides were tested against HT29 CRC cells. Apart from an increase in apoptotic cell population indicated through annexin V-FITC staining, the cyclic dipeptides are known to upregulate the expression of pro-apoptotic markers such as Bid, cytochrome-c, and caspase-3 and to downregulate the antiapoptotic markers such as Bcl-2 [36].

Several unique compounds were obtained through isolation from *Streptomyces* sp. strains from several locations worldwide. Several meroterpenoids, namely, napyradiomycin CNQ525.510B (**55**), napyradiomycin CNQ525.538 (**56**), napyradiomycin CNQ525.538 (**57**), and napyradiomycin CNQ525.600 (**58**), were isolated from an actinomycete strain CNQ525 collected from marine sediment around the coast of La Jolla, California, USA. The MTS assay testing against HCT116 CRC cells showed IC_50_ results of 17, 6, and 49 µM for compounds (**55**), (**56**), and (**58**), respectively. In addition, compound **57** showed weak cytototic activity [37].

Several indole alkaloids were isolated from Streptomyces strain SCSIO 11,791 from the South China Sea, such as dionemycin (**59**), 6-OMe-7′,7″-dichorochromopyrrolic acid (**60**), lynamicin B (**61**), and spiroindimicin B (**62**). These compounds were tested against HCT116 CRC cells using an MTT assay and resulted in IC_50_ values of 4.3, 13.1, 8.7, and 2.2 µM, respectively [38]. The significant suppression of cancer cell viability of compounds (**59**) and (**61**) was correlated with the presence of chlorine atoms on the C-6.

**Figure 4:**
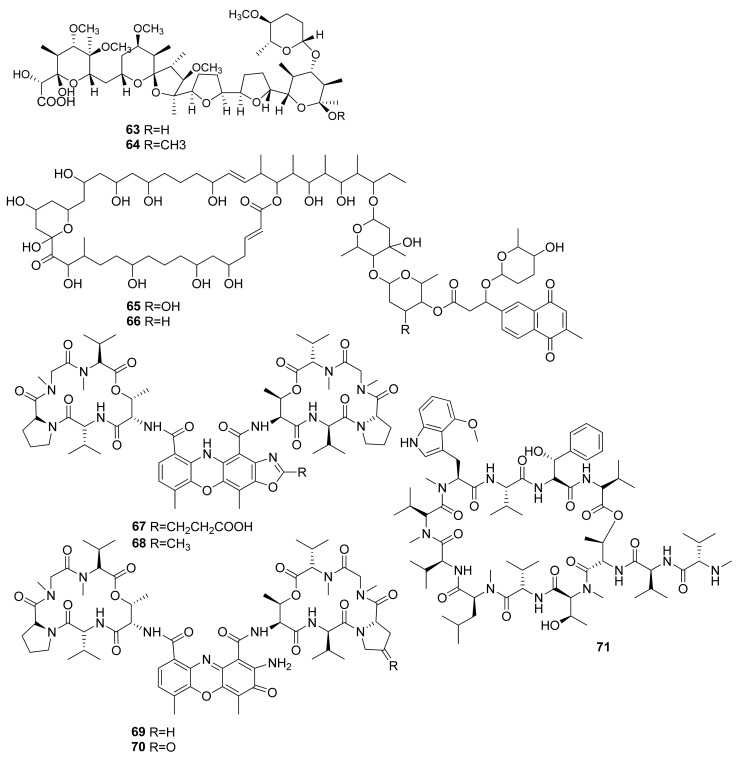
Chemical structures of compounds **63**–**71**.

Two polyether compounds isolated from *Streptomyces cacaoi* 14CM034 were obtained from a sediment sample in Mersin Coastline, Turkey, at a depth of 8 m. K41 A (**63**) and compound **64** were tested through a WST-1 assay against Caco-2 cells and resulted in IC_50_ values of 7.4 and 27.9 µM, respectively (Figure 4). The activity of compound **63** is related to its structure–activity relationship, which has a highly oxygenated (*O*-methyl substituted) polyether framework and the presence of 4′-*O*-methyl-α-d-amicetose (sugar) [39].

**Figure 5:**
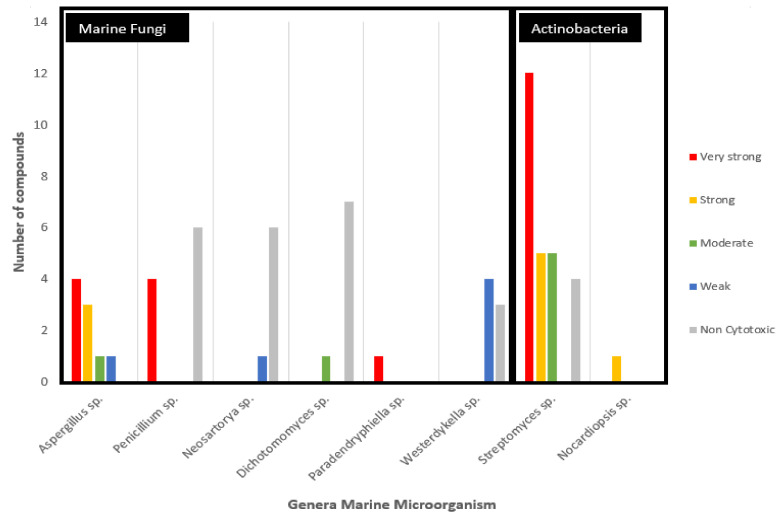
Anticancer potential of secondary metabolites sourced from marine fungi grouped on the basis of the genus of the source microorganism and activity against colorectal cancer cell lines.

Several polyhydroxyl macrolides isolated from *Streptomyces caniferus* GUA-06-05-006A were obtained from around Guadalupe Island, Pacific Ocean. Compounds PM100117 (**65**) and PM100118 (**66**) (Figure 5) were tested against HT29 cells through SRB assay, which resulted in LC_50_ values of 3.8 and 4.1 µM [40]. The marine *Streptomyces* sp. IMB094 isolated from marine sediment in Heishijiao Bay, Dalian, China, resulted in four compounds, namely neo-actinomycin A (**67**), neo-actinomycin B (**68**), actinomycin D (**69**), and actinomycin X_2_ (**70**). Neo-actinomycin A (**67**) showed a very potent cytotoxic effect when tested against HCT116 CRC cells through SRB assays, with an IC_50_ of 38.7 nM, which was 800 times more potent than its positive control, the agent actinomycin D (**69**) [41].

The activity of compound (**67**) might be due to the presence of a carboxyl group that can attack the DNA of colon cancer cells. More importantly, with compound (**67**), there is an absence of planarity of its tetracyclic chromophore, the distinct 5H-oxazolo[4,5-b]phenoxazine part of its structure, and the presence of a 2-amino group. These groups are thought to interact with the DNA of the affected CRC cells, disrupting the structure through the addition of hydrogen bonds bound to their cytosine subunits [41].

The cytotoxic effects of a cyclic peptide isolated from *Streptomyces* sp. SNJ042, ohmyungsamycin A (**71**), was tested through an SRB assay against HCT116 cells and resulted in an IC_50_ value of 7.61 µM, with the positive control etoposide giving an IC_50_ value of 0.52 µM. In terms of mechanisms, the study reported that compound **71** activity against HCT116 cells was through the caspase-mediated apoptosis, as indicated by the increase in sub-G1 phase cells in a dose-dependent manner. Moreover, compound **71** increased the cleavage of PARP by caspase activity. In a concentration-dependent manner, was also correlated with the decreased expression in Skp2, an oncogenic regulator overexpressed in tumor cells, and the increased expressions of p21 and p27, which are CDK inhibitors that function for G0/G1 cell cycle arrest [42].

## 3. Cytotoxicity Assays

In general, cytotoxicity assays are generally carried out using spectrophotometric method with stains such as 3-[4,5-dimethylthiazol-2-yl]-2,5 diphenyl tetrazolium bromide (MTT), 3-(4,5-dimethylthiazol-2-yl)-5-(3-carboxymethoxyphenyl)-2-(4-sulfophenyl)-2H-tetrazolium (MTS), monosodium salt (WST-1), and sulforhodamine B (SRB) [43]. Most of the cytotoxicity assays of metabolites derived from marine fungi are performed comprising of the MTT assay (Table 1), with a few notably highlighted in a few papers utilizing the SRB assay. In contrast, MTS and WST-1 were utilized by only one paper in this review. The MTT assay is preferable to other methods because it arguably has a high reproducibility rate, is sufficiently safe and straightforward to use in laboratory conditions, is highly sensitive for cytotoxicity testing [44], and is suitable for high-throughput screening and miniaturization [45]. 

MTS is considered an enhanced tetrazolium reagent. In the MTS assay, it is not required to solubilize the precipitate of formazan, hence creating a more straightforward protocol [43]. However, unlike the MTS assay, the MTT assay does not have problems when the absorbance is disturbed by cell type/amounts or incubation time, for example, with the presence of 10% albumin in a serum sample [46]. WST-1 is also a tetrazolium reagent in a salt form. Therefore, the WST-1 assay produced formazan, which is highly soluble in water. Similar to the MTS assay, the WST-1 assay also does not require solubilization of formazan as in the MTT assay [47]. The WST-1 assay is very sensitive; however, the reagent is much more expensive than the MTT reagent. In addition to that, the WST-1 assay is heavily affected by the interference of manganese particles and carbon nanotubes [47].

Compared with the other two tetrazolium reagents, MTT is considerably more affordable and, hence, more preferable. Regardless of being preferable, the MTT assay has also disadvantages. The results obtained from the MTT assay can be affected by contaminants’ interference in reading absorbance values. There is also the need for organic solvents such as dimethyl sulfoxide to solubilize resulting crystals. These disadvantages, in turn, can create false positives and false negatives [48]. Nevertheless, these disadvantages are systematically manageable, for example, with proper training against contaminant interference.

Another colorimetric assay for cancer cell viability shown to be in frequent use, albeit fewer, is the SRB assay. Unlike the MTT assay, the SRB assay does not distinguish between viable and dead cells [49]. However, it is also considered a sensitive and rapid method with the principle of the SRB agents binding to amino acid residues in the presence of trichloroacetic acid. It has high reproducibility and linearity and has less likelihood to be affected by environmental factors. The SRB assay is rarely affected by the interference of some compounds. However, multiple washing and drying steps are required. Moreover, homogeneity in tested cancer cell suspensions is necessary [49]. 

## 4. Perspectives

The analyzed anticancer potential compounds are clustered in eight different genera (Figure 5). Larger groupings of the potential marine fungi are observed to belong to *Aspergillus* sp. and *Penicillium* sp., albeit these two genera are shown to be promising in terms of the potency of their compounds, with an equal number (four) of metabolites with very strong potency. Particularly, compound (**29**) isolated from the sole *Paradendryphiella* sp. is considered very strong based on the rules of anticancer potency classification for CRC, as shown in Table 1. Furthermore, it showed high cytotoxic against MCF7-Sh-WISP2 compared with MCF7 or 3T3-F442A [28]. It is a epipolythiodiketopiperazine (ETP) alkaloid and has been known for its anti-fungal and antibacterial activities [52]. This compound was also isolated from marine fungi *Penicillium turbatum* and the *Hyalodendron* sp [53]. Meanwhile, secondary metabolites isolated from *Streptomyces* sp. show the most potent metabolites from actinobacteria. 

The secondary metabolites could be grouped on the basis of their chemical classifications and similarity. In the principle of its basic structure, an indole comprises a benzene ring combined with a pyrrole. This compound and its derivatives have been explored for many pharmacological effects, such as celiac disease, irritable bowel syndrome, and Crohn’s disease (National Center for Biotechnology Information, n.d.). The cytotoxic effects of some of its derivatives can be correlated with the halogenation of surrounding functional groups [38,54]. Moreover, the presence of a chlorine atom-based functional group shows increasing anticancer potency of an indole-based agent. This is explained by the increased cellular suppression of AKT1 kinase, a well-known cell viability intermediator in P13K pathways of colon cancer and other cancers due to the addition of the 3-chloroacetyl group [55].

In another research, the addition of a (3-chloro-2-nitrobenzene)sulfonyl group on an indole-based scaffold compound exhibited a 100-times increment in its potency against cancer cells. This is related to its apoptotic effects, which is similar to the effects by allosteric regulation while also augmenting the stability of the agent [56]. The significant suppression of cancer cell viability is correlated with the presence of chlorine atoms on the C-6″ and shown by compounds **59** and **61** isolated from *Streptomyces* strain SCSIO 11791. These two compounds showed cytotoxicity against MDA-MB-231 human breast adenocarcinoma cells, NCI-H460 human non-small-cell lung cancer cells, HCT-116 colon cancer cells, HepG2 liver cancer cells, and the non-cancerous MCF10A human breast epithelial cells. Moreover, compounds **59** and **61** revealed antibacterial activity against Gram-positive bacteria including *Micrococcus luteus* ML01, *Staphylococcus aureus* ATCC 29213, and a panel of MRSA isolated from human patients (i.e., MRSA 991, MRSA 1862, MRSA 669 A, and MRSA A2) and pigs (MRSA GDQ6P012P and MRSA GDE4P037P) and against Gram-negative bacteria including *Acinetobacter baumannii* ATCC 19606, *Vibrio coralliilyticus* ATCC BAA-450, and *Vibrio alginolyticus* XSBZ14 [38]. A previous study by McArthur et al., demonstrated that compound **61** isolated from a marine actinomycete namely *Marinispora* sp. showed active against drug-resistant pathogens such as methicillin-resistant *Staphylococcus aureus* and *Enterococcus faecium* [57]. This finding may prove using SAR studies that the chlorine atom at C-6 could be pivotal for biological activities in drug design. 

This study noted that the effects of cyclic peptides are very interrelated with various apoptotic markers. Importantly, these are specific to the type and extent of derivatization of the cyclic peptides. The development and optimization of cyclic peptides structures as the basis of drug discovery is essential to aim for higher success of target inhibition ensured by more cyclic peptide hits [58]. For instances, actinomycin D (**69**) is previously well known to possess broad-spectrum antibiotic activities. In a clinical study, it was used as an anticancer drug to treat many tumors, such as Wilms and Ewing tumors, testicular cancer, sarcomas, and choriocarcinoma [59]. In a recent study by Wang et al., comparative anticancer activities of actinomycin D (**69**) and Neo-actinomycins A (**67**) indicated the main activity of 5H-oxazolo[4,5-b]phenoxazine chromophore. The results showed 800-fold reduction in cytotoxic activity relative to **69** against HCT-116 and A549 cancer cells. This could be explained by the activity of the 5H-oxazolo[4,5-b]phenoxazine part of compound **67** on the cytosine subunits of HCT-116 and A-549 cancer cells’ DNA [41].

The use of therapeutic peptides can be preferable for CRC treatment. This is related to their potential characteristics such as higher specificity, lower toxicity, and fewer side effects [60]. Nevertheless, their potential use still faces several limitation regarding their instability against metabolism, poor permeability, solubility, and bioavailability. The more advance research and development are required in order to improve their characteristics, especially on the structure–activity relationship.

## 5. Conclusions

There are 71 secondary metabolites isolated from marine fungi and actinobacteria reviewed in this study, which are summarized as around 44 and 27 metabolites, respectively. The fungi are represented by genera *Aspergillus* sp., *Penicillium* sp., *Neosartorya* sp., *Dichotomomyces* sp., *Paradendryphiella* sp., and *Westerdykella* sp. In contrast, the actinobacteria are represented by genera *Streptomyces* sp. and *Nocardiopsis* sp. Secondary metabolites from marine microorganisms show potential cytotoxic agents on CRC, particularly those sourced from the fungi of *Aspergillus* sp., *Penicillium* sp., and *Paradendryphiella* sp. and from the actinobacteria *Streptomyces* sp. Most of the compounds are found to be indole alkaloids and diketopiperazines (Table 1). The peptides and alkaloids from 71 secondary metabolites are the groups with the most potential for the novelty related to cytotoxic activity. Specifically, these are diketopiperazines (cyclic peptide) for peptides and indoles for alkaloids. Their high potency can be explicated as the effects of the introduction of halogen-based functional groups in indole alkaloids and the presence of sulfide groups in diketopiperazines. It is expected that this provide more insight into accelerating drug discovery research and development, particularly with a focus on CRC drugs and marine microbiology.

## Figures and Tables

**Table 1:** Summary of potential cytotoxic metabolites from various marine microorganisms against colorectal cancer.

**Compound**	**Chemical Class**	**Producing Strain**	**Method, Cell Lines**	Anticancer Potential *	**Ref**
**Marine fungi**					
Rosellichalasin (**1**)	Alkaloid, Cytochalasins	*Aspergillus* sp. nov. F1	*MTT assay, RKO*	Moderate with IC_50_ = 37.3 µM	[16]
Cytochalasin E (**2**)	Alkaloid, Cytochalasins	*Aspergillus* sp. nov. F1	*MTT assay, RKO*	Weak with IC_50_ = 62.3 µM	[16]
Allianthrone A (**3**)	Bianthrone	*A. alliaceus* (new strain, G4)	*MTT assay, HCT116*	Very strong with IC_50_ = 9 µM	[18]
Allianthrone B (**4**)	Bianthrone	*A. alliaceus* (new strain, G4)	*MTT assay, HCT117*	Very strong with IC_50_ = 10.5 µM	[18]
Allianthrone C (**5**)	Bianthrone	*A. alliaceus* (new strain, G4)	*MTT assay, HCT118*	Strong with IC_50_ = 13.7 µM	[18]
Fellutamide F (**6**)	Peptide, Lipopeptide	*A. versicolor* PF10M	*SRB assay, HCT15*	Strong with IC_50_ = 0.13 µg/mL	[19]
Fellutamide C (**7**)	Peptide, Lipopeptide	*A. versicolor* PF10M	*SRB assay, HCT15*	Strong with IC_50_ = 1.74 µg/mL	[19]
Asperphenin A (**8**)	Peptide, Lipopeptidyl Benzophenones	*A. versicolor* Ppf48	*SRB assay, RKO*	Very strong with IC_50_ = 0.84 µM	[22]
Asperphenin B (**9**)	Peptide, Lipopeptidyl Benzophenones	*A. versicolor* Ppf48	*SRB assay, RKO*	Very strong with IC_50_ = 1.26 µM	[22]
Penipacid A (**10**)	Anthranilic acid derivatives	*P. paneum* SD-44	*MTT assay, RKO*	Very strong with IC_50_ = 8.4 µM	[23]
Penipacid B (**11**)	Anthranilic acid derivatives	*P. paneum* SD-44	*MTT assay, RKO*	Non-cytotoxic	[23]
Penipacid C (**12**)	Anthranilic acid derivatives	*P. paneum* SD-44	*MTT assay, RKO*	Non-cytotoxic	[23]
Penipacid D (**13**)	Anthranilic acid derivatives	*P. paneum* SD-44	*MTT assay, RKO*	Non-cytotoxic	[23]
Penipacid E (**14**)	Anthranilic acid derivatives	*P. paneum* SD-44	*MTT assay, RKO*	Very strong with IC_50_ = 9.7 µM	[23]
Compound (**15)**	Anthranilic acid derivatives	*P. paneum* SD-44	*MTT assay, RKO*	Non-cytotoxic	
Brocazine A (**16**)	Peptide, Diketopiperazines with disulfide-bridged	*P. brocae* MA-231	MTT assay, SW480	Very strong with IC_50_ = 2.0 nM	[25]
Brocazine B (**17**)	Peptide, Diketopiperazines with disulfide-bridged	*P. brocae* MA-231	MTT assay, SW480	Very strong with IC_50_ = 1.2 nM	[25]
Brocazine C (**18**)	Peptide, Diketopiperazines with disulfide-bridged	*P. brocae* MA-231	MTT assay, SW480	Non-cytotoxic	[25]
Brocazine D (**19**)	Peptide, Diketopiperazines with disulfide-bridged	*P. brocae* MA-231	MTT assay, SW480	Non-cytotoxic	[25]
Brocazine E (**20**)	Peptide, Diketopiperazines with disulfide-bridged	*P. brocae* MA-231	-	Not tested but showed activity against Du145, Hela, HepG2, MCF-7, NCI-H460, SGC-7901, SW1990, and U251	[25]
Brocazine F (**21**)	Peptide, Diketopiperazines with disulfide-bridged	*P. brocae* MA-231	-	Not tested but showed activity against Du145, Hela, HepG2, MCF-7, NCI-H460, SGC-7901, SW1990, and U251	[25]
18-oxo-19,20-dihydrophomacin C (**22**)	Alkaloid, Cytochalasans	*W. dispersa* XL602	*MTT assay, HT29*	Non-cytotoxic	[27]
18-oxo-19-methoxy-19,20- dihydrophomacin C C (**23**)	Alkaloid Cytochalasans	*W. dispersa* XL602	*MTT assay, HT29*	Non-cytotoxic	[27]
18-oxo-19-hydroxyl-19,20-dihydrophomacin C (**24**)	Alkaloid, Cytochalasans	*W. dispersa* XL602	*MTT assay, HT29*	Non-cytotoxic	[27]
19,20-dihydrophomacin C (**25**)	Alkaloid, Cytochalasans	*W. dispersa* XL602	*MTT assay, HT29*	Weak with IC_50_ = 49.09 µM	[27]
19-methoxy-19,20-dihydrophomacin C (**26**)	Alkaloid, Cytochalasans	*W. dispersa* XL602	*MTT assay, HT29*	Weak with IC_50_ = 55.31 µM	[27]
19-hydroxyl-19,20-dihydrophomacin C (**27**)	Alkaloid, Cytochalasans	*W. dispersa* XL602	*MTT assay, HT29*	Weak with IC_50_ = 55.48 µM	[27]
Gymnastatin Z (**28**)	Alkaloid, Tyrosine-derivative	*W. dispersa* XL602	*MTT assay, HT29*	Weak with IC_50_ = 49.31 µM	[27]
(3R, 6R) Hyalodendrin (**29**)	Heterocyclic aromatics, Piperazine	*P. salina* PC 362H	*MTT assay, HCT116oxa*	Very Strong with IC_50_ = 25.7 nM	[28]
Dichotomocej A (**30**)	Amides	*D. cejpii* F31-1	*SRB assay, HCT116*	Non-cytotoxic	[30]
Diorcinol (**31**)	Polyphenols	*D. cejpii* F31-1	*SRB assay, HCT116*	Non-cytotoxic	[30]
3-*O*-methyldiorcinol (**32**)	Polyphonols	*D. cejpii* F31-1	*SRB assay, HCT116*	Non-cytotoxic	[30]
Butyl (2-ethylhexyl) phthalate (**33**)	Phthalic Acid Esters	*D. cejpii* F31-1	*SRB assay, HCT116*	Non-cytotoxic	[30]
Dichocerazine A (**34**)	Diketopiperazines	*D. cejpii* F31-1	*SRB assay, HCT116*	Non-cytotoxic	[30]
Pityriacitrin (**35**)	Alkaloid, Indoles	*D. cejpii* F31-1	*SRB assay, HCT116*	Moderate with IC_50_ = 35.1 µM	[30]
Stellarine A (**36**)	Alkaloid, Indoles	*D. cejpii* F31-1	*SRB assay, HCT116*	Non-cytotoxic	[30]
Indolyl-3-acetic acid methyl ester (**37**)	Alkaloid, Indoles	*D. cejpii* F31-1	*SRB assay, HCT116*	Non-cytotoxic	[30]
Chevalone C (**38**)	Meroterpenoids	*N. siamensis* KUFA 0017	*MTT assay, HCT116*	Non-cytotoxic with IC_50_ = 153 µM	[31]
Nortryptoquivaline (**39**)	α-amino acid ester derivatives	*N. siamensis* KUFA 0017	*MTT assay, HCT116*	Non-cytotoxic with IC_50_ = 114 µM	[31]
Tryptoquivaline H (**40**)	α-amino acid ester derivatives	*N. siamensis* KUFA 0017	*MTT assay, HCT116*	Non-cytotoxic with IC_50_ = 202 µM	[31]
Fiscalin A (**41**)	Alkaloid, Indoles	*N. siamensis* KUFA 0017	*MTT assay, HCT116*	Non-cytotoxic with IC_50_ = 123 µM	[31]
*epi*-Fiscalin A (**42**)	Alkaloid, Indoles	*N. siamensis* KUFA 0017	*MTT assay, HCT116*	Non-cytotoxic with IC_50_ = 277 µM	[31]
*epi*-Neofiscalin A (**43**)	Alkaloid, Indoles	*N. siamensis* KUFA 0017	*MTT assay, HCT116*	Non-cytotoxic with IC_50_ = 203 µM	[31]
*epi*-Fiscalin C (**44**)	Alkaloid, Indoles	*N. siamensis* KUFA 0017	*MTT assay, HCT116*	Weak with IC_50_ = 86 µM	[31]
**Actinobacteria**					
Androsamide (**45**)	Peptide, Cyclic Tetrapeptide	*Nocardiopsis sp.* CNT-189	*MTT assay, Caco-2* and *HCT116*	Strong (for both cell lines tested) with IC_50_ = 13 µM againts Caco-2 cells and IC_50_ = 21 µM againts HCT116 cells	[32]
Cyclo(Pro-Ala) (**46**)	Peptide, Diketopiperazines	*S. nigra sp.* nov. 452	*MTT assay, HCT116*	Moderate with IC_50_ = 47.6 µg/mL	[33]
Cyclo(Pro-Val) (**47**)	Peptide, Diketopiperazines	*S. nigra sp.* nov. 452	*MTT assay, HCT116*	Moderate with IC_50_ = 67.2 µg/mL	[33]
Cyclo(Pro-Leu) (**48**)	Peptide, Diketopiperazines	*S. nigra sp.* nov. 452	*MTT assay, HCT116*	Moderate with IC_50_ = 92.6 µg/mL	[33]
Cyclo(Pro-Phe) (**49**)	Peptide, Diketopiperazines	*S. nigra sp.* nov. 452	*MTT assay, HCT116*	Moderate with IC_50_ = 32.3 µg/mL	[33]
Furan-type Compound (**50**)	Heterocyclic aromatics, Furan	*Streptomyces sp.* VN1	*MTT assay, HCT116*	Non-cytotoxic with IC_50_ = 123.7 µM	[34]
Petrocidin A (**51**)	Peptide, Cyclic Dipeptide	*Streptomyces* sp. SBT348	*MTT assay, HT29*	Strong with IC_50_ = 5.3 µg/mL	[35]
2,3-dihydroxybenzoic acid (**52**)	Benzene	*Streptomyces* sp. SBT348	*MTT assay, HT29*	Non-cytotoxic	
2,3-Dihydroxybenzamide (**53**)	Benzene	*Streptomyces* sp. SBT348	*MTT assay, HT29*	Strong with IC_50_ = 3.8 µg/mL	[35]
Maltol (**54**)	4*H*-pyran	*Streptomyces* sp. SBT348	*MTT assay, HT29*	Non-cytotoxic	
Napyradiomycin CNQ525.510B (**55**)	Terpene, Meroterpenoids	*Streptomyces sp.* CNQ525	*MTS assay, HCT116*	Strong with IC_50_ = 17 µM	[37]
Napyradiomycin CNQ525.538 (**56**)	Terpene, Meroterpenoids	*Streptomyces sp.* CNQ525	*MTS assay, HCT116*	Very strong with IC_50_ = 6 µM	[37]
Napyradiomycin CNQ525.554 (**57**)	Terpene, Meroterpenoids	*Streptomyces sp.* CNQ525	*MTS assay, HCT116*	Non-cytotoxic	
Napyradiomycin CNQ525.600 (**58**)	Terpene, Meroterpenoids	*Streptomyces sp.* CNQ525	*MTS assay, HCT116*	Moderate with IC_50_ = 49 µM	[37]
Dionemycin (**59**)	Alkaloid, Indoles	*Streptomyces* strain SCSIO 11791	*MTT assay, HCT116*	Very strong with IC_50_ = 4.3 µM	[38]
6-OMe-7′,7″-dichorochromopyrrolic acid (**60**)	Alkaloid, Indoles	*Streptomyces* strain SCSIO 11791	*MTT assay, HCT116*	Strong againts with IC_50_ = 13.1 µM	[38]
Lynamicin B (**61**)	Alkaloid, Indoles	*Streptomyces* strain SCSIO 11791	*MTT assay, HCT116*	Very strong with IC_50_ = 8.7 µM	[38]
Spiroindimicin B (**62**)	Alkaloid, Indoles	*Streptomyces* strain SCSIO 11791	*MTT assay, HCT116*	Very strong with IC_50_ = 2.2 µM	[38]
K41 A (**63**)	Polyether	*S. cacaoi* 14CM034	*WST-1 assay, Caco-2*	Very Strong with IC_50_ = 7.4 µM	[39]
Compound (**64**)	Polyether	*S. cacaoi* 14CM034	*WST-1 assay, Caco-2*	Strong with IC_50_ = 7.4 µM	
PM100117 (**65**)	Macrolide, Polyhydroxyl	*S. caniferus* GUA-06-05-006A	*SRB assay, HT29*	Very Strong with LC_50_ = 3.8 µM	[40]
PM100118 (**66**)	Macrolide, Polyhydroxyl	*S. caniferus* GUA-06-05-006A	*SRB assay, HT29*	Very Strong with IC_50_ = 4.1 µM	[40]
Neo-actinomycin A (**67**)	Peptide, Cyclic Dipeptide	*Streptomyces sp.* IMB094	*SRB assay, HCT116*	Very Strong with IC_50_ = 38.7 nM	[41]
Neo-actinomycin B (**68**)	Peptide, Cyclic Dipeptide	*Streptomyces sp.* IMB094	*SRB assay, HCT116*	Very Strong with IC_50_ = 339.1 nM	[41]
Actinomycin D (**69**)	Peptide, Cyclic Dipeptide	*Streptomyces sp.* IMB094	*SRB assay, HCT116*	Very Strong with IC_50_ = 0.045 nM	[41]
Actinomycin X_2_ (**70**)	Peptide, Cyclic Dipeptide	*Streptomyces sp.* IMB094	*SRB assay, HCT116*	Very Strong with IC_50_ = 0.0075 nM	[41]
Ohmyungsamycin A (**71**)	Peptide, Cyclic Dipeptide	*Streptomyces strain* SNJ042	*SRB assay, HCT116*	Very Strong with IC_50_ = 7.61 µM	[42]

* IC_50_ values in μg/mL are <21 μg/mL for strong, 21–200 μg/mL for moderate, and 201–500 μg/mL for weak cytotoxicity, with values >501 μg/mL as non-cytotoxic [50]. IC50 measurements in μM, values of 1–10 μM can be defined as very strong, 11–25 μM defined as strong, 26–50 μM defined as moderate, and 51–100 μM defined as weak cytotoxic. Values above 100 μM are considered non-cytotoxic [51].
